# Phase IV, Open-Label, Safety Study Evaluating the Use of Dexmedetomidine in Pediatric Patients Undergoing Procedure-Type Sedation

**DOI:** 10.3389/fphar.2017.00529

**Published:** 2017-08-11

**Authors:** Edmund H. Jooste, Gregory B. Hammer, Christian R. Reyes, Vaibhav Katkade, Peter Szmuk

**Affiliations:** ^1^Pediatric Cardiac Anesthesiology, Duke Children's Hospital and Health Center Durham, NC, United States; ^2^Departments of Anesthesiology, Perioperative and Pain Medicine and Pediatrics, Stanford University School of Medicine Stanford, CA, United States; ^3^Department of Biostatistics, Pfizer Manila, Philippines; ^4^Department of Medical Affairs, Pfizer Collegeville, PA, United States; ^5^Department of Anesthesiology and Pain Medicine, Children's Health Medical Center, University of Texas Southwestern Medical Center Dallas, TX, United States; ^6^Outcomes Research Consortium Cleveland, OH, United States

**Keywords:** alpha_2_ agonist, dexmedetomidine, non-intubated, pediatric, sedation, NCT01519167; https://clinicaltrials.gov/ct2/show/NCT01519167

## Abstract

Dexmedetomidine (Precedex™) may be used as an alternative sedative in children, maintaining spontaneous breathing, and avoiding tracheal intubation in a non-intubated moderate or deep sedation (NI-MDS) approach. This open-label, single-arm, multicenter study evaluated the safety of dexmedetomidine in a pediatric population receiving NI-MDS in an operating room or a procedure room, with an intensivist or anesthesiologist in attendance, for elective diagnostic or therapeutic procedures expected to take at least 30 min. The primary endpoint was incidence of treatment-emergent adverse events (TEAEs). Patients received one of two doses dependent on age: patients aged ≥28 weeks' gestational age to <1 month postnatal received dose level 1 (0.1 μg/kg load; 0.05–0.2 μg/kg/h infusion); those aged 1 month to <17 years received dose level 2 (1 μg/kg load; 0.2–2.0 μg/kg/h infusion). Sedation efficacy was assessed and defined as adequate sedation for at least 80% of the time and successful completion of the procedure without the need for rescue medication. In all, 91 patients were enrolled (dose level 1, *n* = 1; dose level 2, *n* = 90); of these, 90 received treatment and 82 completed the study. Eight patients in dose level 2 discontinued treatment for the following reasons: early completion of diagnostic or therapeutic procedure (*n* = 3); change in medical condition (need for intubation) requiring deeper level of sedation (*n* = 2); adverse event (AE; hives and emesis), lack of efficacy, and physician decision (patient not sedated enough to complete procedure; *n* = 1 each). Sixty-seven patients experienced 147 TEAEs. The two most commonly reported AEs were respiratory depression (bradypnea; reported per protocol-defined criteria, based on absolute respiratory rate values for age or relative decrease of 30% from baseline) and hypotension. Four patients received glycopyrrolate for bradycardia and seven patients received intravenous fluids for hypotension. SpO_2_ dropped by 10% in two patients, but resolved without need for manual ventilation. All other reported AEs were consistent with the known safety profile of dexmedetomidine. Two of the 78 patients in the efficacy-evaluable population met all sedation efficacy criteria. Dexmedetomidine was well-tolerated in pediatric patients undergoing procedure-type sedation.

## Introduction

General anesthesia is often utilized in children who undergo diagnostic and painful therapeutic procedures. Dexmedetomidine (Precedex™) is an alpha_2_ adrenergic receptor agonist with sympatholytic, sedative, analgesic, and anxiolytic effects that attenuate the catecholamine response to perioperative stress (Ebert et al., 2000; Kamibayashi and Maze, 2000). As a sedative alternative, dexmedetomidine may be used to achieve moderate or deep sedation, wherein spontaneous breathing is maintained and tracheal intubation is avoided in a non-intubated moderate or deep sedation (NI-MDS) approach. Dexmedetomidine has not been associated with respiratory depression when used alone, even during deep sedation (Ebert et al., 2000; Venn et al., 2000). It has been used successfully in pediatric patients for sedation during and after mechanical ventilation, for treating the clinical signs and symptoms of drug withdrawal, and to prevent post-operative shivering, and is well-tolerated in this patient population (Tobias, 2000; Tobias et al., 2003; Berkenbosch et al., 2005; Chrysostomou et al., 2006; Teshome et al., 2014; Ahmed et al., 2015; Malhotra et al., 2016). Despite its use in pediatric clinical practice, dexmedetomidine is not approved for use in children by the US Food and Drug Administration (FDA). Clinicians should also be aware of the adverse events associated with the use of dexmedetomidine, which include, but are not limited to various hemodynamic changes such as biphasic blood pressure response, bradycardia, and reduced respiratory rate (Hammer et al., 2008; Mason et al., 2008).

The objective of this study was to evaluate the safety of dexmedetomidine in a pediatric population receiving NI-MDS. The study was conducted to fulfill the requirements of the Pediatric Research Equity Act (PREA) after the approval of Precedex™ for procedural sedation in adults.

## Methods

### Study design

**Figure 1:**
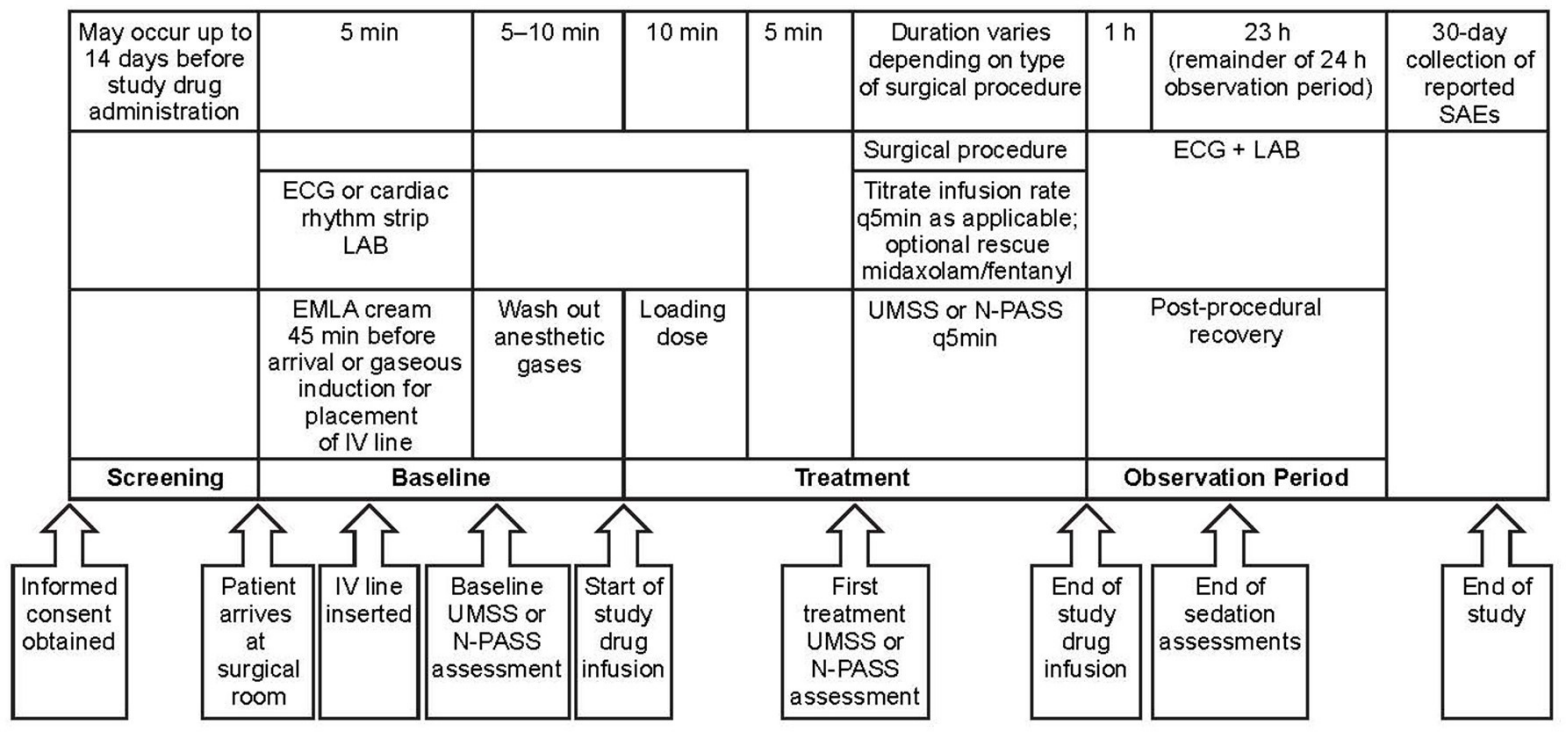
Study design. One patient enrolled in dose level 2 did not receive study medication due to administrative reasons. EMLA, eutectic mixture of local anesthetics; ECG, electrocardiogram; IV, intravenous; LAB, laboratory assessment; N-PASS, Neonatal Pain, Agitation and Sedation Scale; q5 min, every 5 min; SAE, serious adverse event; UMSS, University of Michigan Sedation Scale.

This open-label, single-arm, multicenter study (Figure 1) was carried out in a pediatric population receiving NI-MDS in an operating room or a procedure room, with an intensivist or anesthesiologist in attendance, for elective diagnostic or therapeutic procedures expected to take at least 30 min to perform. The trial is registered on ClinicalTrials.gov (NCT01519167).

The study was carried out in accordance with the recommendations of the International Conference on Harmonization guidelines, guidelines governing clinical study conduct, and the ethical principles derived from the Declaration of Helsinki. An Independent Ethics Committee/Institutional Review Board [Copernicus Group Independent Review Board (IRB)] approved the protocol, and each of the study sites subsequently had the protocol approved by their own IRB committees (Duke University Health System IRB Office; IRB Stanford University; Human Research Subjects Protection Office, University of Puerto Rico Medical Science Campus; Vanderbilt University Human Research, Copernicus Group IRB three sites: University of Pittsburgh Medical Center, Lone Peak Oral and Maxillofacial Surgery, Anaheim Clinical Trials, LLC; Human Subject Research Office, University of Miami; University of Arkansas for Medical Sciences IRB; University of Texas Southwestern Medical Center IRB). Written informed consent was obtained from parents and assent was obtained from patients aged ≥10 years. Patients were enrolled at eight sites between October 4, 2012 and January 13, 2014. Owing to the unique characteristics of the pediatric population, and for ethical reasons, a placebo-controlled study design was not considered. The duration of the study was ~45 days [enrollment (which could take place up to 14 days prior to the study day), 1 study day, and a 30-day follow-up period for reported serious adverse events (SAEs)].

### Study eligibility

Inclusion criteria included: gestational age ≥28 weeks and age <17 years; American Society of Anesthesiologists (ASA) Physical Status I, II, or III, scheduled for elective non-invasive diagnostic/therapeutic procedure, elective minimally invasive diagnostic/therapeutic procedure, or elective surgical procedure. Female patients of childbearing potential were required to have a negative pregnancy test.

Exclusion criteria were: weight <1,000 g; general anesthesia within 7 days; exposure to dexmedetomidine within 48 h; administration of an intravenous (IV) opioid within 1 h, oral/intramuscular opioid within 4 h, any pre-induction medication (i.e., ketamine, chloral hydrate, benzodiazepines) within 4 h, or an alpha_2_ agonist or antagonist within 14 days prior to study drug administration; use of an endotracheal tube or laryngeal mask airway, epidural, or spinal anesthesia; contraindication to dexmedetomidine, opiates, benzodiazepines, or other alpha_2_ agonists. Patients were also excluded if they had the following: bradycardia or hypotension immediately before dosing; second- or third-degree heart block at screening or baseline (presence of a temporary or permanent pacemaker waived this criterion); acute febrile illness with a temperature (core or tympanic) ≥38°C; moderate to severe sleep apnea; acute myocardial infarction diagnosed by confirmatory laboratory findings within 6 weeks of screening; or oxygen saturation (SpO_2_) ≤ 90% at screening or baseline (except for patients with known cyanotic heart disease undergoing cardiac catheterization).

### Patient population

Patients representing three age groups and three populations were enrolled. The age groups were: group 1, ≥28 weeks' gestational age to <3 years; group 2, ≥3 years to <12 years; and group 3, ≥12 years to < 17 years. The procedural populations were: group I, non-invasive diagnostic/therapeutic procedures including ultrasound (US), computed tomography (CT) scans, magnetic resonance imaging, cardiac catheterization, or transthoracic echocardiogram; group II, minimally invasive diagnostic/therapeutic procedures including minimally invasive procedures performed under US or CT guidance (e.g., US- or CT-guided solid organ biopsy), and routine myocardial biopsies in cardiac transplant recipients; group III, superficial surgical procedures (e.g., excisions, biopsies).

### Study endpoints

#### Safety

The primary safety endpoint was incidence of treatment emergent adverse events (TEAEs). Secondary safety endpoints were: incidence of TEAEs in patients undergoing diagnostic/therapeutic procedures compared with those undergoing surgical procedures; incidence of hypotension, hypertension, bradycardia, and tachycardia compared with baseline; incidence of protocol-defined respiratory depression [defined either as absolute thresholds by age (Table 1) or as relative thresholds, i.e., decreases from baseline of >30% in respiratory rate and 10% in SpO_2_; end-tidal capnography = 0 for more than 30 s]; respiratory depression requiring intervention; time from discontinuation of study drug to suitability for discharge from the recovery room, defined as an Aldrete score ≥9 (Aldrete, 1995); and anesthesiologist assessment of pain perception by patient, ease of maintenance, hemodynamic and respiratory stability, and patient co-operation. Hemodynamic stability was defined by maintenance of systolic blood pressure (SBP) and heart rate, each within ±30% of pre-study drug baseline.

**Table 1:** Absolute thresholds for respiratory rate defining AE of respiratory depression.

**Age**	**Respiratory rate (breaths per minute)**
≥28 weeks' gestational age to <1 month	<40
1 month to <3 months	<35
3 months to <6 months	<30
6 months to <1 year	<25
1 year to <3 years	<20
3 years to <6 years	<20
6 years to <12 years	<14
12 to 17 years	<12

#### Efficacy

The primary efficacy endpoint was the rate of success in sedation, which was defined as a patient having all of the following: (i) adequate level of sedation [University of Michigan Sedation Scale (UMSS) score, (Malviya et al., 2002) between 1 and 3, or Neonatal Pain, Agitation and Sedation Scale (N-PASS) score, (Hummel et al., 2008) between −5 and −2] for at least 80% of the time during administration of study drug; and (ii) successful completion of procedure without a need for rescue sedation (midazolam); And (iii) undergone the procedure without artificial ventilation or intervention to restore baseline or normal hemodynamic status. Subjects were given an UMSS/N-PASS score every 5 min for the first 30 min, every 15 min during the study drug administration, and again every 5 min for the first 15 min upon arrival in the recovery room, then every 15 min until they met criteria for discharge from the recovery room were met. An UMSS/N-PASS score was also obtained prior to administration of any rescue midazolam or fentanyl.

Secondary efficacy endpoints were: incidence of patients without need for artificial ventilation or intervention to restore baseline or normal hemodynamic status; incidence of patients not requiring rescue midazolam; time from onset of study drug infusion to first dose of rescue midazolam; frequency of rescue midazolam; total amount of rescue midazolam; frequency (number of patients requiring at least one instance) of rescue fentanyl from the start of IV sedation to completion of the procedure; total amount of rescue fentanyl required; and incidence of patients who converted to alternative sedation and/or anesthetic therapy due to failure of treatment with study drug and rescue medication.

### Assessments

#### Safety

At screening, a complete medical history was obtained and physical examination and 12-lead electrocardiogram were performed. Temperature was monitored at baseline and on arrival in the recovery room. At baseline, three distinct measurements (3 min apart) of blood pressure and heart rate were obtained to determine average baseline values. SBP and diastolic blood pressure (DBP), SpO_2_, heart rate, and respiratory rate were measured immediately before administration of the loading dose, every 5 (±1) min for the first 30 min of dexmedetomidine infusion, and then every 15 (±5) min until the end of dexmedetomidine infusion. Following dexmedetomidine administration, vital signs were collected every 5 (±1) min for the first 15 min, then every 15 (±5) min while in the recovery room, until discharge. Continuous capnography was performed during the procedure using a nasal cannula. Absence of a waveform on end tidal carbon dioxide value for 30 s qualified as an AE. Chemistry and hematology samples were obtained for laboratory tests. Immediately after the patient was transferred to the recovery room, the anesthesiologist rated the ease of maintenance of appropriate intraoperative sedation level, respiratory stability, hemodynamic stability, and patient co-operation using an anesthesiologist assessment questionnaire.

#### Efficacy

Prior to the start of dexmedetomidine infusion, a baseline score on the UMSS (Malviya et al., 2002) or N-PASS (Hummel et al., 2008) was obtained. A UMSS or an N-PASS score was also obtained prior to the administration of any rescue midazolam. The Aldrete Scoring System (Aldrete, 1995) was used for all patients upon arrival in the recovery room and every 15 min thereafter while in the recovery room.

### Treatment

#### Study treatment

All patients received dexmedetomidine administered as a two-stage infusion. At dose level 1, the patient aged at least ≥28 weeks' gestational age to <1 month postnatal age received a loading dose of 0.1 μg/kg, administered over 10 min followed by a maintenance dose, starting at 0.1 μg/kg/h with titration to between 0.05 and 0.2 μg/kg/h as indicated to achieve and/or maintain an N-PASS score ≤ −2. At dose level 2, patients aged 1 month to <17 years received a loading dose of 1 μg/kg administered over 10 min followed by a maintenance dose, starting at 0.6 μg/kg/h with titration to between 0.2 and 2.0 μg/kg/h as indicated to achieve and/or maintain a UMSS score ≥1. Dexmedetomidine was infused using a controlled infusion device and could not be delivered as a fast bolus or IV push. Sedation dosages were calculated for each patient using their most recent weight measurements prior to commencement of dexmedetomidine.

#### Rescue therapy

Rescue medications employed were midazolam for sedation and fentanyl for analgesia, both of which are considered standards of care. Administration of rescue medication was not permitted during the first 15 min of study drug administration.

### Statistical analyses

An estimated 90 patients were planned to be enrolled, with at least 25 patients for each age group and each procedural population, to allow for an adequate description of AEs and other safety outcomes (such as hemodynamic effects). Statistical analyses were performed using SAS version 9.1 (SAS Institute Inc., Cary, NC). Descriptive statistics for continuous data included number of patients, mean, standard deviation (SD), median, minimum, first quartile (Q1), third quartile (Q3), and maximum. Coefficient of variation was also provided as appropriate. Descriptive statistics for categorical data were recorded as count and percent.

## Results

### Patient disposition and baseline characteristics

**Figure 2:**
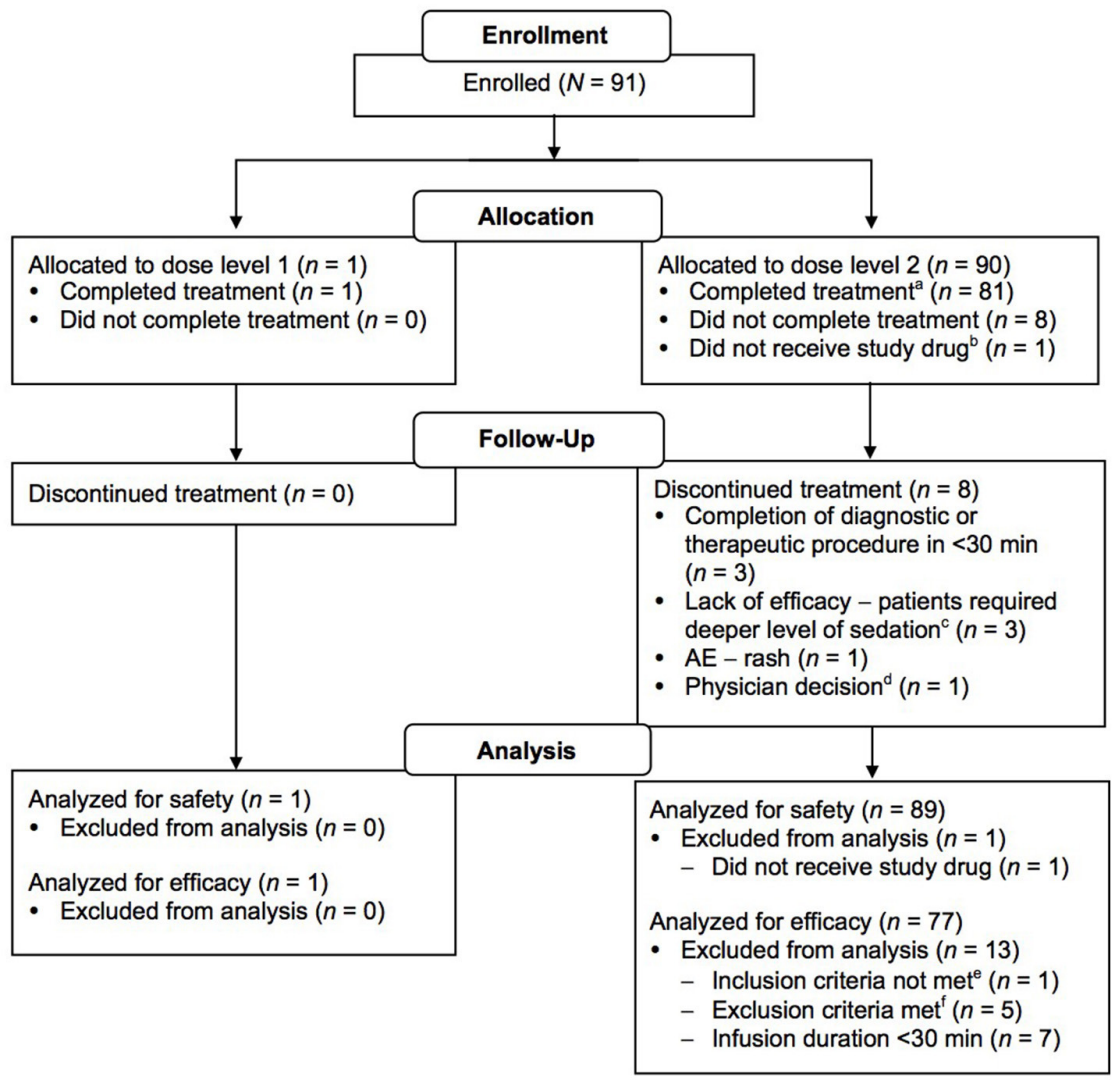
CONSORT diagram. ^a^Patients who received study drug infusion for at least 30 min. ^b^Patient was not treated due to administrative reasons (approval to prepare study drug could not be obtained within the 2-week period between enrollment and treatment). ^c^Patients unable to be sedated adequately with protocol medications; converted to propofol sedation. ^d^Error in drug administration and clamp never released, no drug reached patient. Physician proceeded with alternative sedation. ^e^Patient was <17 years of age. ^f^Three patients had hypotension immediately before dosing; two patients had participated in an experimental/investigational drug study within 30 days prior to study drug administration.

Ninety-one patients were enrolled (*n* = 1, dose level 1; *n* = 90, dose level 2); of these, 90 received treatment and 82 completed treatment (having received at least 30 min of drug infusion; *n* = 1, dose level 1; *n* = 81, dose level 2; Figure 2). One of the patients enrolled in dose level 2 did not receive study medication due to administrative reasons. Patient enrollment by age group and procedure type is shown in Table 2. Patient demographic and baseline characteristics for the safety evaluable population are shown in Table 3. Most patients [*n* = 88 (97.8%)] had reported a previous medical history or concurrent disease; those most commonly reported were in the following systems: head, eyes, ears, nose and throat, and neurological [*n* = 35 (38.9%) each], gastrointestinal [*n* = 32 (35.6%)], and respiratory and musculoskeletal [*n* = 31 (34.4%) each]. Eight patients in dose level 2 discontinued treatment for the following reasons: early completion of diagnostic or therapeutic procedure (*n* = 3); change in medical condition requiring deeper level of sedation (*n* = 2); AE, lack of efficacy, and physician decision (*n* = 1 each). The mean durations of loading and maintenance doses were 10.0 and 55.8 min, respectively. Total doses were 0.267 μg/kg in dose level 1 and 2.521 μg/kg in dose level 2.

**Table 2:** Patient enrollment by age group and type of procedure.

**Procedure type**	**Age**			
	**≥28 weeks' gestation to <3 years**	**≥3 years to <12 years**	**≥12 years to <17 years**	**Total**
Non-invasive diagnostic/therapeutic	23	18	5	46
Minimally invasive diagnostic/therapeutic	6	10	16^a^	32
Surgical	0	6	7	13
Total	29	34	28	91

a *One patient was aged 17 years at the time of enrollment*.

**Table 3:** Demographic and baseline characteristics (safety evaluable population).

**Characteristic**	**Total (*N* = 90)**
**Race**, ***n*** **(%)**	
African–American	14 (15.6)
Asian	1 (1.1)
Black	9 (10.0)
White	60 (66.7)
Other	6 (6.7)
**Sex**, ***n*** **(%)**	
Female	41 (45.6)
Male	49 (54.4)
**Ethnicity**, ***n*** **(%)**	
Hispanic or Latino	23 (25.6)
Not Hispanic or Latino	67 (74.4)
Age, years, mean (range)	7.8 (0.1, 17.3)
Height, cm, mean (SD)	121.4 (38.6)
Weight, kg, mean (SD)	35.7 (28.7)
**ASA Physical Status**, ***n*** **(%)**	
I (Normal healthy subject)	16 (17.8)
II (Patient with mild systemic disease)	63 (70.0)
III (Patient with severe systemic disease)	11 (12.2)
**Type of procedure**, ***n*** **(%)**	
Non-invasive diagnostic/therapeutic	46 (51.1)
Minimally invasive diagnostic/therapeutic	31 (34.4)
Surgical	13 (14.4)

*ASA, American Society of Anesthesiologists; SD, standard deviation*.

### Safety outcomes

All patients who received study medication (*n* = 90) were included in the safety evaluable population. To be able to describe clinically relevant findings and to help the reader differentiate between an isolated non-clinically relevant decrease in respiratory rate and actual respiratory depression, the authors have used the word “bradypnea” to describe the former, and “respiratory depression” for the latter. Sixty-seven patients (74.4%) experienced 147 TEAEs. The one patient in dose level 1 experienced one TEAE, and 66 patients in dose level 2 reported 146 TEAEs. The TEAE in dose level 1 was reported only because the infant's baseline respiratory rate [30 beats per minute (bpm)] was below the absolute threshold defined by the protocol (40 bpm), i.e., it was not due to a clinically relevant change from baseline. The two most commonly reported AEs were bradypnea (reported per protocol-defined criteria, based on absolute respiratory rate values for age, or a relative decrease of 30% from baseline) and hypotension (SBP decrease of >30% from baseline; Table 4). None of the patients required manual ventilation for respiratory depression; furthermore, the concomitant use of midazolam and fentanyl was provided as alternative etiology by investigators for several of these events. The majority of TEAEs (137 in 58 patients) were mild in intensity. Eight patients experienced nine TEAEs that were of moderate intensity [hypotension (*n* = 3); respiratory depression (*n* = 2); vomiting, headache, agitation, and hypertension (*n* = 1 each)]. One patient experienced one TEAE (hyperkalemia) that was of severe intensity, although the concomitant use of enalapril was suggested as an alternative etiology. The most commonly reported TEAEs (reported in ≥2 patients) were: hypotension in 30 patients; bradypnea in 19 patients; bradycardia in three patients; and vomiting, decreased SBP, headache, and respiratory depression (in two patients each). During the post-treatment period, two patients reported nausea, and one reported vomiting. With the exception of one patient with hyperkalemia, no other clinically relevant change was observed in laboratory values. No relevant changes in physical examination or ECGs were observed.

**Table 4:** Summary of treatment-emergent adverse events.

	**Dose level 1 (*N* = 1)**	**Dose level 2 (*N* = 89)**	**Total (*N* = 90)**
Number of events	1	146	147
Number of patients with at least one event	1 (100.0)	66 (74.2)	67 (74.4)
Cardiac rhythm disorders	0	5 (5.6)	5 (5.6)
Bradycardia	0	4 (4.5)	4 (4.4)
Tachycardia	0	1 (1.1)	1 (1.1)
Gastrointestinal disorders	0	5 (5.6)	5 (5.6)
Diarrhea	0	1 (1.1)	1 (1.1)
Nausea	0	2 (2.2)	2 (2.2)
Vomiting		2 (2.2)	2 (2.2)
Blood pressure disorders	0	5 (5.6)	5 (5.6)
Decreased DBP	0	2 (2.2)	2 (2.2)
Decreased SBP	0	3 (3.4)	3 (3.3)
Vascular disorders	0	37 (41.6)	37 (41.1)
Hypertension	0	2 (2.2)	2 (2.2)
Hypotension	0	37 (41.6)	37 (41.1)
Metabolism and nutrition disorders	0	2 (2.2)	2 (2.2)
Hyperglycemia	0	1 (1.1)	1 (1.1)
Hyperkalemia	0	1 (1.1)	1 (1.1)
Nervous system disorders	0	5 (5.6)	5 (5.6)
Headache	0	3 (3.4)	3 (3.3)
Somnolence	0	1 (1.1)	1 (1.1)
Syncope	0	1 (1.1)	1 (1.1)
Respiratory disorders	1 (100)	53 (59.6)	54 (60.0)
Respiratory depression (hypoxia)	0	2 (2.2)	2 (2.2)
Bradypnea	1 (100)	53 (59.6)	54 (60.0)
Skin and subcutaneous tissue disorders	0	1 (1.1)	1 (1.1)
Rash	0	1 (1.1)	1 (1.1)

*Subjects were counted once within each system organ class or for each preferred term and may have had more than one AE*.

*DBP, diastolic blood pressure; SBP, systolic blood pressure*.

Only two SAEs were reported. The first was syncope, reported in a 16 year-old, who underwent a renal biopsy. At around 11 p.m. the same evening, the patient got out of bed “felt weird” and had a syncopal event. The nursing staff revived the patient with a sternal rub, after which, the patient was alert and orientated. Vital signs, when taken, were normal. The investigator considered the event was possibly vasovagal and likely not related to dexmedetomidine, as elimination of dexmedetomidine occurs primarily through the urine, with an elimination half-life of ~2 to 2.5 h (Su and Hammer, 2011) and the event occurred more than 3 half-lives after dexmedetomidine was discontinued.

The second SAE was hematuria, which occurred in an 8 year-old who had undergone renal biopsy and reported hematuria 2 days later that required hospital admission. The on-site investigator felt this was most likely caused by the renal biopsy and was not related to dexmedetomidine. Both patients were enrolled in dose level 2 and the SAEs resolved without treatment. One patient in dose level 2 experienced two TEAEs (vomiting and rash) that led to discontinuation of study drug. These were assessed by the investigator as possibly related to dexmedetomidine and resolved after the patient discontinued the drug. No SAEs resulted in death.

### Secondary safety endpoints

#### Incidence of TEAEs between procedures

TEAEs were more commonly reported in patients undergoing surgical procedures (92.3%) compared with those undergoing minimally invasive (74.2%) or non-invasive/therapeutic procedures (69.6%).

#### Respiratory and hemodynamic changes from baseline

For the one neonate enrolled in dose level 1, the maximum noted decreases from baseline in heart rate and mean arterial pressure (MAP) were −18 bpm and −20 mmHg, respectively. For respiratory rate, the maximum increase and decrease from the baseline of 30 breaths/min were were +7 and −3 breaths/min, respectively. In dose level 2, decreases from baseline of ~10 bpm in heart rate and ~3 mmHg in MAP were noted. As captured through vital signs (Table 4), four subjects received glycopyrrolate for bradycardia and seven subjects received IV fluid boli for hypotension; all had resolution of clinical signs. Two incidences of respiratory depression occurred, where the Sp0_2_ dropped below 10% from baseline; both resolved without the need for manual ventilation. On average, patients were outside the pre-defined hemodynamic range (i.e., SBP and heart rate within 30% of baseline) 8% of the time at most in the treatment period and 15% of the time at most in the post-treatment period. Changes in heart rate were more frequent than those for SBP in both the treatment and post-treatment periods. At least half of the patients in the safety population had no time outside the pre-defined hemodynamic range in either the treatment or post-treatment period.

#### Time to discharge

Overall, the median time from discontinuation of study drug to discharge (Aldrete score ≥9) was 63 min (95% confidence interval [CI]: 51, 70). Patients undergoing surgical procedures required more time for discharge than those undergoing minimally invasive or non-invasive procedures (73, 69, and 50.5 min, respectively).

#### Anesthesiologist assessment

The mean score for ease of maintaining sedation on a Visual Analogue Scale (0 = not difficult, to 10 = extremely difficult) was 3.43. Similarly, the mean scores for hemodynamic and respiratory stability (0 = very stable, to 10 = extremely unstable) were 1.01 and 0.79, respectively, and for patient co-operation (0 = very co-operative, to 10 = extremely un-co-operative) was 2.83.

### Efficacy outcomes

This study was primarily a safety study and efficacy variables in the context of the design features, were supportive only. All patients who received the study drug for at least 30 min and had no major protocol deviations (*n* = 78) were included in the efficacy population. Success in sedation was defined as having an adequate level of sedation (UMSS score 1 to 3 or N-PASS score −5 to −2) for at least 80% of the time during administration of study drug, and successful completion of the procedure without the need for rescue sedation; and having undergone the procedure without artificial ventilation or intervention to restore baseline or normal hemodynamic status. Two of 78 (2.6%) patients in the efficacy-evaluable population fulfilled all the criteria for having success in sedation per the protocol-defined criteria. Both patients were in the non-invasive procedures group and received dexmedetomidine at dose level 2.

Rescue midazolam during study drug infusion was not required in 19 (24.4%) patients. More patients who underwent non-invasive procedures (13 of 41 [31.7%]) had no requirement for rescue midazolam compared with other procedural groups (minimally invasive procedures, five of 25 [20.0%]; surgical procedures, one of 12 [8.3%]). In the efficacy-evaluable population, no patients required artificial ventilation and most patients (69 of 78 [88.5%]) underwent procedures without intervention to restore baseline or normal hemodynamic status. Interventions that were given were fluid replacement (*n* = 7) and glycopyrrolate (*n* = 4). Thirty of the 78 (38.5%) patients in the efficacy evaluable population were adequately sedated at least 80% of the time during study drug infusion. In the non-invasive procedures group, 51.2% of patients were adequately sedated for 80% of the time, in contrast with 16.7% in the surgical procedures group. The median time to first dose of rescue midazolam from onset of study drug infusion in the efficacy-evaluable population was 22 min (95% CI: 17, 25). The time to first use of rescue medication was similar across the three types of procedures, although slightly longer in the non-invasive procedures group (24 [95% CI: 16, 30] min) compared with the minimally invasive (20 [95% CI: 17, 34] min) and surgical (20 [95% CI: 15, 25] min) procedures groups. However, the study protocol mandated that use of rescue medication was not permitted during the first 15 min of study drug administration.

From the start of IV sedation to completion of the procedure, 59 of 78 (75.6%) patients required rescue midazolam. The median frequency of usage of midazolam in the rescued patients was 1 (range: 1–4) and the mean total amount of midazolam received was 2.28 mg (SD: 1.312). Overall, the frequency and dose used was similar across the three types of procedures (Table 5). In total, 40 of 78 (51.2%) patients required at least one instance of rescue fentanyl for analgesia from the start of IV sedation to completion of the procedure. The median frequency of fentanyl usage in rescued patients was 1.5 (range: 1–5) and the mean total amount of fentanyl received was 67.10 μg (SD: 68.84). The type of procedure influenced the amount of analgesia required, in increasing order both in frequency and dose from non-invasive to surgical procedures (Table 5). Four (4.4%) of the 90 treated patients required conversion to a different anesthetic therapy: one in the non-invasive procedures group, one in the minimally invasive procedures group, and two in the surgical procedures group. In all, 22 (24.4%) patients received a local or regional anesthetic block; in half of these cases (*n* = 11), bupivacaine was used as the local anesthetic.

**Table 5:** Summary of rescue therapy (efficacy evaluable population for rescued patients).

	**Dose level 1 (*N* = 1)**	**Dose level 2 (*N* = 77)**	**Total (*N* = 78)**
**FREQUENCY OF MIDAZOLAM USE, MEDIAN (MIN, MAX)**			
Non-invasive diagnostic/therapeutic procedures	0	2 (1–4)	2 (1–4)
Minimally invasive diagnostic/therapeutic procedures	N/A	1 (1–2)	1 (1–2)
Surgical procedures	N/A	1 (1–2)	1 (1–2)
**TOTAL AMOUNT OF MIDAZOLAM, mg, MEAN (SD)**			
Non-invasive diagnostic/therapeutic procedures	0	2.62 (1.61)	2.62 (1.61)
Minimally invasive diagnostic/therapeutic procedures	N/A	1.70 (0.77)	1.70 (0.77)
Surgical procedures	N/A	2.45 (0.91)	2.45 (0.91)
**FREQUENCY OF FENTANYL USE, MEDIAN (MIN, MAX)**			
Non-invasive diagnostic/therapeutic procedures	0	1 (0–3)	1 (1–3)
Minimally invasive diagnostic/therapeutic procedures	N/A	1 (1–5)	1 (1–5)
Surgical procedures	N/A	2.50 (1–5)	2.50 (1–5)
**TOTAL AMOUNT OF FENTANYL**, μ**g, MEAN (SD)**			
Non-invasive diagnostic/therapeutic procedures	0	10.83 (8.01)	10.83 (8.01)
Minimally invasive diagnostic/therapeutic procedures	N/A	61.86 (65.74)	61.86 (65.74)
Surgical procedures	N/A	104.83 (72.01)	104.83 (72.01)

*N/A, not applicable; SD, standard deviation*.

## Discussion

In order to evaluate the safety of dexmedetomidine during NI-MDS, an open-label study was conducted in infants and children aged ≥28 weeks' gestation to <17 years. Only one patient was enrolled in dose level 1 owing to the challenges associated with sedating non-intubated subjects aged <1 month. Dexmedetomidine was found in general to be safe and was associated with few clinically significant respiratory or hemodynamic changes. It was shown not be an effective single agent in this setting but the study was not powered to evaluate efficacy, the efficacy outcomes reported are supportive only in the context of the design features of this safety study.

All reported AEs were consistent with the known safety profile of dexmedetomidine (Constantin et al., 2016). In general, there was an association between the degree of invasiveness of the procedure, increased use of rescue sedation and analgesia therapy, and increased frequency of bradypnea and hypotension TEAEs. In the authors' opinion, the high incidence of the protocol-defined decrease in respiratory rate of >30% from baseline is not clinically significant and represents a normal decrease in the respiratory rate of a child who is calmly sedated compared with that of a child in a relatively anxious pre-sedation state. Furthermore, the 2 events of >10% decrease in SpO_2_ from baseline resolved quickly and without the need for mechanical ventilation. Previous comments have been made about the potential for prolonged time in the post-anesthesia care unit with the use of higher doses of dexmedetomidine (Ni et al., 2015). Time to discharge in the present study (50–73 min) appears to be in line with other pediatric discharge times reported in the literature (45–157 min) (Picard et al., 2000; Moncel et al., 2015).

Based on the composite efficacy endpoint used in this study, dexmedetomidine as a single agent showed no evidence of success in sedating pediatric patients undergoing procedure-type sedation; only 2.6% of patients in the efficacy-evaluable population fulfilled the criteria for having success in sedation. The composite endpoint may have been too stringent to allow the assessment of a clinically meaningful benefit of dexmedetomidine in children. Furthermore, in this study, dexmedetomidine was initially administered as a single agent, whereas in clinical practice, it is most often used in combination with other agents (Buck, 2010). Indeed, in the authors' experience, current daily practical use of dexmedetomidine is very different to that followed in this study, i.e., the load is given in a manner more like an IV push, instead of over 10 min, and the infusion rates can be higher than the maximum of 2 μg/kg/h specified in the current study.

Concern for neuroprotection is an important consideration with the use of anesthesia in pediatric patients, especially in children younger than 3 years. Preclinical studies have demonstrated that dexmedetomidine does not cause neurotoxicity (Brummett et al., 2008; Koo et al., 2014; Li et al., 2016). Therefore, when used as an adjunct therapy, it has the potential to reduce the required dose and/or duration of other agents and anesthetics known to be neurotoxic. In a study of 50 pediatric patients undergoing heart surgery, a combination of dexmedetomidine and low-dose fentanyl (15 μg/kg) had the same anesthetic effect as a 30 μg/kg dose of fentanyl (Sun et al., 2015). The results of a meta-analysis that compared use of dexmedetomidine with placebo or other alternative anesthetic agents during pediatric congenital heart disease surgery suggested that dexmedetomidine was associated with shorter length of mechanical ventilation, lower post-operative fentanyl and morphine requirements, reduced stress response, and lower risk of delirium (Pan et al., 2016). Similarly, in a retrospective comparison in pediatric patients with congenital heart disease requiring post-operative sedation, patients who received dexmedetomidine required significantly lower doses of adjunctive sedative/analgesic drugs compared with those who received midazolam (Jiang et al., 2015).

## Study limitations

This study has several limitations. It was designed to satisfy PREA requirements after the approval of Precedex™ for procedural sedation in adults (Bergese et al., 2010; Candiotti et al., 2010); therefore, to ensure alignment with the previous study design in adult participants, there were limitations on the design of the current study. The use of baseline vital signs criteria to compare sedated intra- and post-treatment vital signs resulted in over-reporting of AEs; most notably, all of the reported decreases in respiratory rate were deemed not clinically significant. Also, limited dosing regimens were mandated (e.g., low doses, a long bolus time, and long intervals between allowable dose changes), which resulted in suboptimal sedation and, consequently, necessitated a greater use of rescue medication. Furthermore, dexmedetomidine is generally used as a part of a multimodal regimen because monotherapy is often inadequate for painful procedures (Grewal, 2011); as such, its lack of efficacy in this study was not unexpected. If the protocol had permitted the use of usual premedication or pre-emptive analgesics in the procedures, the efficacy success rate may have been much improved. Finally, the enrollment of only one patient in the dose level 1 (neonatal) group makes it impossible to draw any conclusions for this population and dosing regimen. Furthermore, it was difficult to enroll younger and sicker patients into the cohorts, which likely had an impact on the low incidence of AE.

## Conclusion

Dexmedetomidine was well-tolerated in pediatric patients undergoing procedure-type sedation. No major changes in SpO_2_ were observed, no interventions were required to preserve respiratory function either during or post study drug infusion, and none of the patients required artificial ventilation. All other reported AEs were consistent with the known safety profile of dexmedetomidine. Although further well-designed, controlled studies may be needed to adequately evaluate its efficacy in pediatric patients, dexmedetomidine may be considered as a sedation/anesthetic option for this patient population.

## Author contributions

EJ, GH, and PS were study investigators, participated in all stages of recruitment of the patients and critically reviewed the manuscript. CR was responsible for analysis of the data and critically reviewed the manuscript. VK critically reviewed the manuscript. All authors read and approved the final manuscript.

### Conflict of interest statement

CR and VK are employees of Pfizer and have Pfizer stock options. This study was conducted and funded by Hospira to fulfill the requirements for the Pediatric Research Equity Act (PREA) after the approval of Precedex™ for procedural sedation in adults. The other authors declare that the research was conducted in the absence of any commercial or financial relationships that could be construed as a potential conflict of interest.

## Abbreviations

ASA: American Society of Anesthesiologists
NI-MDS: non-intubated moderate or deep sedation
N-PASS: Neonatal Pain, Agitation and Sedation Scale
PREA: Pediatric Research Equity Act
UMSS: University of Michigan Sedation Scale.
